# Supplementary material for: Public Dental Service Access Policies for People with Autism Spectrum Disorder (ASD) in Salvador, Bahia, Brazil: A Pre-Evaluation Study
Source: Int J Environ Res Public Health. 2024 Apr 27;21(5):555. doi: 10.3390/ijerph21050555 (PMC11120991; doi:10.3390/ijerph21050555)
Supplement: Supplementary file 1 [file ijerph-21-00555-s001.zip › ijerph-2942189-supplementary.pdf]

Chart S1. List of documents analyzed and summary of contents.

N	Document title	Publication year	Source	Summary
D1	The Brazilian Federal Constitution	1988	Brazilian Federal Government	In Brazil, health is a right of all citizens and a duty of the state. Access to healthcare is guaranteed to every citizen, and the state is responsible for providing health services, resources, and public policies to improve the population's well-being.
D2	The Child and Adolescent statute	1990	Brazilian Federal Government	The Child and Adolescents Statute is a comprehensive legal framework to protect the rights of children and adolescents in Brazil. It protects children and adolescents in situations of vulnerability, including disabilities, allocates resources for social assistance programs, and establishes specific measures to safeguard the rights of vulnerable groups.
D3	National Oral Health Policy	2004	Brazilian Federal Government	The Brazilian Oral Health Policy acknowledges the importance of addressing the oral health needs of people with disabilities as part of its commitment to equity and inclusivity in healthcare. The State must ensure that people with disabilities have access to appropriate oral health services.
D4	Convention on the Rights of Persons with Disabilities	2006	United Nations (UN)	The UN CRPD is an international treaty that aims to protect and promote the rights and dignity of persons with disabilities. It advocates that people with disabilities should have access to all health goods and services without discrimination.
D5	National Plan for People with Disabilities – Living without Limits	2011	Brazilian Federal Government	The National Plan for People with Disabilities represents a coordinated effort to advance the rights and well-being of persons with disabilities and to create a more inclusive and accessible society for all
D6	Care Network for People with Disabilities	2012	Brazilian Federal Government	The care network for people with disabilities is a network of services and support systems to provide holistic care and assistance, encompassing various healthcare facilities, social services, educational institutions, and community organizations working together to address the diverse needs of this specific group.

D7	National Policy for the Protection of the Rights of People with Autism Spectrum Disorders (Berenice Piana Law – 12.764)	2012	Brazilian Federal Government	Law 12.764/2012 addresses the needs and rights of individuals with ASD in Brazil. It promotes people with ASD inclusion, well-being, and full participation in society.
D8	Guidelines for the Rehabilitation of People with Autism Spectrum Disorders (ASD)	2014	Brazilian Federal Government	This document results from efforts by professionals, researchers, and specialists in various health professions and from scientific and professional societies, as well as representatives from civil society. It offers guidelines to the multi-professional teams of the SUS and families of ASD individuals about the flows for monitoring and caring for people with ASD in the Brazilian Health System network.
D9	Care Pathway for People with Autism Spectrum Disorders and their Families in the Psychosocial Care Network of the Unified Health System	2016	Brazilian Federal Government	This document provides for the CEOs' participation in the care network for people with disabilities. It is up to the municipal administration to adhere to the strategy or not. The CEOs with this strategy must provide a dental chair with a service of at least 40 hours a week exclusively for people with disabilities. The role of PHC in dental care is unclear in this document.
D10	Referral Protocol for Oral Health Specialties in Salvador	2017	Salvador City Hall	People with ASD should enter the public health system through PHC, which can refer them to Dental Specialty Centers (CEOs) as necessary, which happens most of the time, primarily through the Center for Attention to People with Disabilities (NAPES, in Portuguese, Núcleo de Atenção a Pacientes Especiais)
D11	Oral Health in the Unified Health System	2018	Brazilian Federal Government	This book systematizes previous documents on clinical guidance in oral health in the SUS. It suggests referral and counter-referral protocols for primary care and for the Dental Specialties Center (CEO), as well as including an approach to Patients with Special Needs, hitherto not addressed in any Ministry of Health material, including people with Autism Spectrum Disorder (ASD).
D12	Guide to Oral Health Care for People with Disabilities 2019	2019	Brazilian Federal Government	Offers guidance to professionals in the Unified Health System (SUS) Care Network, promoting the qualification of dental care based on strategies for management and care in a qualified and safe manner, in the logic of integrality and humanization and in accordance with standards established by scientific evidence

D13	The Municipal Health Plan 2018-2021	2018	Salvador City Hall	The Municipal Health Plan presents actions that reinforces the Referral Protocol for Oral Health Specialties in the Municipality of Salvador, referring to PHC, Dental Specialty Centers (CEOs) and Hospitals

Figure S1: Model of the consent form

TERMO DE CONSENTIMENTO LIVRE E ESCLARECIDO

Prezado (a) senhor (a), sou Ednaldo de Jesus Filho, vinculado ao Doutorado em Odontologia e Saúde da Faculdade de Odontologia da Universidade Federal da Bahia (FOUFBA), e estou realizando juntamente com as professoras Maria Cristina Teixeira Cangussu (FOUFBA) e Sandra Garrido de Barros (FOUFBA) o estudo intitulado "TRANSTORNO DO ESPECTRO AUTISTA E SAÚDE BUCAL". Gostaríamos de convida-lo (a) a participar desta pesquisa, como voluntário. Esta pesquisa tem como objetivo conhecer as condições de saúde bucal em pessoas com Transtorno do Espectro Autista, bem como avaliar a política e as condições de acesso e utilização dos serviços de odontologia oferecidos pelo SUS a estas pessoas. Este trabalho foi aprovado pelo Comitê de Ética e Pesquisa da Faculdade de Odontologia da Universidade Federal da Bahia. Os procedimentos adotados obedecem a resolução n. 466/12 do Conselho Nacional de Saúde. Os pesquisadores se responsabilizam em manter a sua identidade em sigilo, utilizando as informações somente para fins acadêmicos. Poderão a qualquer tempo solicitar esclarecimentos referentes a todas as etapas desta pesquisa, pessoalmente, por telefone ou endereço eletrônico, conforme dados disponibilizados a seguir: FOUFBA – Faculdade de Odontologia da Universidade Federal da Bahia, Av. Araújo Pinho, nº 62, Canela - Salvador CEP: 40100-150, telefone central - 71:3283-8962; no mesmo endereço localiza-se o Comitê de Ética em Pesquisa da FOUFBA, telefone – 3283-8964; e contato telefônico do pesquisador Ednaldo de Jesus Filho através do número 71-991787371 ou e-mail edodonto@yahoo.com.br. Após estes esclarecimentos, solicitamos a seguir o seu consentimento para a participação do (a) senhor (a) na pesquisa de forma livre e esclarecida.

CONSENTIMENTO LIVRE E ESCLARECIDO: Eu li este formulário de consentimento (ou alguém leu e o explicou para mim), realizei a impressão ou download em PDF ou recebi uma 2ª via deste Termo, todas as minhas dúvidas foram esclarecidas e eu concordo em participar deste estudo. Estou ciente de que posso sair a qualquer momento, sem perder o direito de procurar ajuda dos pesquisadores. Autorizo os pesquisadores a entrarem em contato comigo caso haja necessidade (marque abaixo a opção desejada):
pelo telefone – não ( ) sim ( ) ______________________________________
e-mail – não ( ) sim ( ) ____
pessoalmente. – não ( ) sim ( ) ____________________________________

VOCÊ DESEJA PARTICIPAR DESTA PESQUISA? ( ) Sim ( ) Não desejo participar

__________________________ ________________________ Data: ___/____/______

Nome do participante Assinatura do participante

__________________________ ________________________ Data: ___/____/______

Nome do pesquisador Assinatura do pesquisador

Figure S2: The interview script.

Roteiro para entrevistas com gestores e profissionais (adaptado a posição do entrevistado)

a. Como ocorre o encaminhamento de pessoas com TEA para serviços de saúde bucal no município?
b. Como se dá a relação entre a coordenação de Saúde Bucal e de Saúde Mental no município?
c. Existe ou já existiu algum estudo envolvendo a condição de Saúde Bucal de Pessoas com TEA no município? Em caso positivo, quais os resultados?
d. Existe alguma ferramenta ou indicador que monitore o atendimento odontológico das pessoas com TEA no município de Salvador?
e. Existe alguma ação estratégica voltada para o atendimento odontológico de pessoas com TEA dentro da rede de Atenção à Saúde Bucal no município de Salvador?
f. Como se dá a entrada da pessoa com TEA para o atendimento odontológico na rede de atenção à saúde do município de Salvador? Qual o tempo de espera para a marcação da 1ª consulta? Existe alguma restrição (material/pessoal) para o atendimento de pessoas com TEA no CEO?
g. O município recebe/oferece algum incentivo para o atendimento de pessoas com TEA na rede de Atenção Primária de Saúde no município de Salvador?
h. Os serviços (APS e CEO) sabem informar o quantitativo de pessoas com TEA que estão em atendimento?
i. Descreva as iniciativas do município para ações estratégicas de saúde bucal das pessoas com TEA.
